# Effects of Delayed Cord Clamping on 4-Month Ferritin Levels, Brain Myelin Content, and Neurodevelopment: A Randomized Controlled Trial

**DOI:** 10.1016/j.jpeds.2018.06.006

**Published:** 2018-12

**Authors:** Judith S. Mercer, Debra A. Erickson-Owens, Sean C.L. Deoni, Douglas C. Dean, Jennifer Collins, Ashley B. Parker, Meijia Wang, Sarah Joelson, Emily N. Mercer, James F. Padbury

**Affiliations:** 1 College of Nursing, University of Rhode Island, Kingston, RI; 2 Pediatrics, Alpert School of Medicine, Brown University, Providence, RI; 3 Department of Pediatrics, Women and Infants Hospital of Rhode Island, Providence, RI; 4 Advanced Baby Imaging Lab, Memorial Hospital of Rhode Island, Pawtucket, RI; 5 Department of Radiology, University of Colorado School of Medicine, Aurora, CO; 6 Waisman Center, University of Wisconsin, Madison, WI

**Keywords:** delayed cord clamping, umbilical cord milking, myelin, iron, placental transfusion, DCC, Delayed cord clamping, GLM, General linear model, ICC, Immediate cord clamping, ID, Iron deficiency, mcDESPOT, Multicomponent-Driven Equilibrium Single-Pulse Observation of T1 and T2, MRI, Magnetic resonance imaging, RCT, Randomized controlled trial, RPBV, Residual placental blood volume, VFm, Myelin water volume fraction

## Abstract

**Objective:**

To evaluate whether placental transfusion influences brain myelination at 4 months of age.

**Study design:**

A partially blinded, randomized controlled trial was conducted at a level III maternity hospital in the US. Seventy-three healthy term pregnant women and their singleton fetuses were randomized to either delayed umbilical cord clamping (DCC, >5 minutes) or immediate clamping (ICC, <20 seconds). At 4 months of age, blood was drawn for ferritin levels. Neurodevelopmental testing (Mullen Scales of Early Learning) was administered, and brain myelin content was measured with magnetic resonance imaging. Correlations between myelin content and ferritin levels and group-wise DCC vs ICC brain myelin content were completed.

**Results:**

In the DCC and ICC groups, clamping time was 172 ± 188 seconds vs 28 ± 76 seconds (*P* < .002), respectively; the 48-hour hematocrit was 57.6% vs 53.1% (*P* < .01). At 4 months, infants with DCC had significantly greater ferritin levels (96.4 vs 65.3 ng/dL, *P* = .03). There was a positive relationship between ferritin and myelin content. Infants randomized to the DCC group had greater myelin content in the internal capsule and other early maturing brain regions associated with motor, visual, and sensory processing/function. No differences were seen between groups in the Mullen testing.

**Conclusion:**

At 4 months, infants born at term receiving DCC had greater ferritin levels and increased brain myelin in areas important for early life functional development. Endowment of iron-rich red blood cells obtained through DCC may offer a longitudinal advantage for early white matter development.

**Trial registration:**

ClinicalTrials.gov: NCT01620008.

See editorial, p 8

Alt-text: Unlabelled box

Delayed cord clamping (DCC) at birth supports a transfer of blood from the placenta to the newborn infant, resulting in a 30% increase in blood volume and a 50% increase in iron-rich red cell volume.^1^, ^2^ Ferritin, the major iron storage protein in the body, is increased after DCC through 6 months of age,^3^ whereas immediate cord clamping (ICC) decreases early iron stores^4^, ^5^, ^6^, ^7^, ^8^, ^9^, ^10^, ^11^ and may contribute to iron deficiency (ID) in infancy.^12^ Infant ID can adversely affect cognitive, motor, social–emotional, and behavioral development.^13^, ^14^, ^15^, ^16^, ^17^, ^18^ Red blood cells from DCC may provide a critical early iron endowment for the oligodendrocytes, the most metabolically active cells in the brain. These myelin-producing cells are sensitive to iron deprivation, as oligodendrocytes require iron for both maturation and function.^19^, ^20^, ^21^, ^22^, ^23^, ^24^ Iron is transported readily across the blood–brain barrier, on demand, through the process of transferrin endocytosis.^20^ Studies in animals clearly link hypomyelination with ID and neurodevelopmental impairment,^15^ and abnormal myelination is associated with a variety of childhood developmental disorders, including dyslexia and autism spectrum disorders.^25^, ^26^, ^27^

Based on the importance of iron availability for oligodendrocytes to form myelin, we investigated the potential effects of timing of umbilical cord clamping (DCC vs ICC) on myelin maturation. We employed a novel, noninvasive neuroimaging technique termed mcDESPOT (multicomponent-Driven Equilibrium Single-Pulse Observation of T1 and T2) to quantify myelin water volume fraction (VFm), a surrogate measure for myelin content^28^, ^29^, ^30^ that has been used previously to characterize normative patterns of myelination in healthy infants,^31^, ^32^ and to investigate relationships between myelin content and evolving brain function and cognitive skills.^33^, ^34^

We hypothesized that infants born at term exposed to placental transfusion via DCC (or cord milking) would have greater iron stores and enhanced myelin formation showing increased myelin content at 4 months of age compared with infants who were exposed to ICC.

## Methods

Enrollment for this randomized controlled trial (RCT) was conducted from July 2012 to November 2015 (ClinicalTrials.gov: NCT01620008), and corresponding follow-up at 4 months of age occurred from November 2012 to March 2016. The study was conducted at Women and Infants Hospital of Rhode Island and Brown University (Providence, Rhode Island) after approval by the institutional review boards from Women and Infants Hospital, the University of Rhode Island, and Brown University. Results of the birth and 2-day data have been published previously.^35^ Assessments at 12 months of age were completed in November 2016 and 24-month assessments in December 2017.

### Intervention, Randomization, and Blinding

Methods for enrollment and randomization for this study have been described previously.^35^ We obtained informed consent from healthy, term pregnant women and enrolled them prenatally. Just before birth, blocked stratified randomization was used (in sequenced and sealed envelopes) to assign women to either DCC (>5 minutes) or ICC (<20 seconds). Milking of the cord (5 times) was the proxy for DCC at cesarean delivery or if the provider could not delay. Residual placental blood volume (RPBV), the remaining blood in the placenta after birth, was obtained via drainage.^35^ Blinding of the research assistants at the infant's birth was not possible due to the nature of the intervention. However, group assignment was not revealed to the pediatric or laboratory staff or the magnetic resonance imaging (MRI) and developmental testing personnel. All study staff except the birth research assistants were unaware of the randomization assignment.

### Participant Follow-Up

There were 4 separate data collection points for the subjects at 4 months of age: well-baby visit, blood draw for iron indices (including ferritin), MRI, and neurodevelopmental testing. To support retention, contact with participants was maintained by the research assistants and the lead research nurse. Research assistants attended the infants' 4-month well-baby pediatric visits and collected growth and health data. Within 1 week of the blood draw, MRI scans were completed (limited to 140 days of life for the 4-month analyses). Neurodevelopmental testing was completed within 1 week of a successful MRI.

At 4 months, a heel capillary blood sample was collected for a complete blood count and iron indices including ferritin, transferrin, soluble transferrin receptor, and C-reactive protein. The samples were collected by a pediatric nurse at the child's home or by a laboratory technician at the hospital laboratory. Discussion of the blood sample methods is found in the Appendix (available at www.jpeds.com).

Infants underwent MRI during natural, nonsedated sleep at either nap or bedtime on a Siemens Tim Trio 3 Tesla scanner (Siemens Healthineers Headquarters, Erlangen, Germany). Measures of brain myelin content, as measured by VFm, were acquired from 4-month-old participants using the mcDESPOT MRI technique and following previously described guidelines for infant neuroimaging.^36^ Further details about the MRI technique can be found in the Appendix. Notably, this technique has been used extensively to study myelination patterns in infancy and early childhood.^31^, ^32^, ^33^, ^37^, ^38^

Within 7 days of a successful MRI, each child was assessed with the Mullen Scales of Early Learning, a standardized and population-normed tool for assessing fine and gross motor control, visual reception, and expressive and receptive language for children up to 5 years, 9 months of age.^39^ In addition to individual age-normalized domain scores, there are 3 composite Mullen scores that reflect overall cognitive ability (Early Learning Composite) as well as verbal and nonverbal development quotients. Each of these composite scores is expressed as a standard score with a mean of 100 and an SD of 15. In addition, mothers were asked to complete the Edinburgh Postnatal Depression Scale at the enrollment visit and at 4 months after birth as well as the Parental Stress Index at 4 months of age.

### Sample Size

Effect sizes based on data from previous studies of ferritin levels after DCC suggest that without adjustment sample sizes of 30 per group would have more than 80% power at an alpha of 0.05 to detect differences in ferritin levels between the 2 groups.^3^, ^5^, ^6^ Substantial variance reduction (at least 50%) can be achieved by controlling for baseline covariates, such as age, gestational age, and birth weight, as planned. No previous data exist for the effects of umbilical cord clamping time on VFm. Deoni et al reported that the SD of VFm estimates in white matter is 5% in healthy children.^40^ To reliably measure a 5% VFm difference between the control and experimental groups, using a 2-sample *t* test (alpha = 0.05, power = 0.80), 16 observations per group were required.

### Statistical Analyses

Data analyses included 2-sided Pearson χ^2^ tests, 2-sample *t* tests, and Wilcoxon rank-sum tests for non-normally distributed variables. Primary analyses were conducted using intention-to-treat, and sensitivity analyses were conducted using actual treatment to assess the robustness of the findings and to examine results of the biological variables. Log transformation was used for the analysis of the ferritin levels due to non-normal distribution of the ferritin data. The level of significance was .05 (2-tailed) for main effects. Data were analyzed with SAS 9.3 (SAS Institute, Inc, Cary, North Carolina) and SPSS Version 23 (IBM Corp, Armonk, New York).

### Image Analysis and Statistical Testing

Associations between VFm and 4-month blood ferritin levels were evaluated at each image voxel using a general linear model (GLM) that included age, gestational age, and birth weight as additional variables of noninterest. Voxel-wise VFm differences between the DCC and ICC groups additionally were investigated by performing an unpaired *t* test. The FMRIB Software Library package (FMRIB Analysis Group, Oxford, United Kingdom) was used to construct the GLM, and both the GLM and group differences were tested nonparametrically using permutation testing (randomize) and 5000 permutations. Significance was defined as *P* < .05, with correction for the multiple comparisons in MRI data using a cluster-based technique.^41^, ^42^

## Results

**Figure 1:**
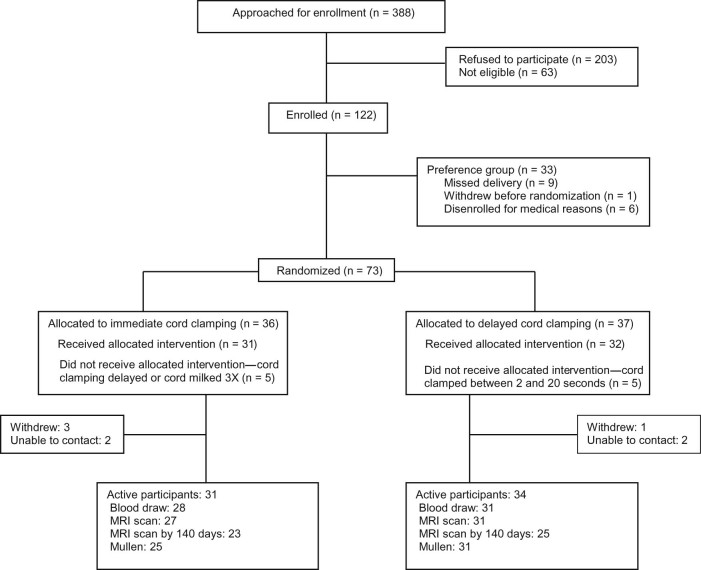
Infant brain study 4-month randomized cohort flowchart.

Seventy-three healthy term pregnant women were randomized to DCC or ICC. At 4 months, 64 (88%) infants were active participants. Of those, 59 (92%) had blood draws and 58 (91%) underwent MRI scanning. Fifty-six (88%) infants completed the developmental testing. Of the 58 MRIs completed, 48 MRIs were completed before 140 days (83%) and 44 (92%) were usable (Figure 1; available at www.jpeds.com). Only data from these 44 infants are reported here and are referred to as the MRI cohort.

Participant demographics and clinical variables for infants with an MRI within 140 days are shown in Tables I and II. There were no significant group differences with respect to maternal age, education, type of insurance, mode of delivery, gestational age, birth weight, or sex. Consistent with the previous report,^35^ infants in the MRI cohort with DCC had longer cord-clamping time (per protocol) (*P* = .002), less RPBV at birth (*P* = .05), and greater hematocrit levels at 2 days of age (*P* = .01). There was no difference in cord ferritin levels between the groups.

**Table I:** Maternal and infant demographics and clinical variables at birth (for infants who were successfully scanned at 4 months, intention-to-treat)

Characteristics	DCC	ICC	*P* value
	(n = 23)	(n = 21)	
Maternal			
Age, y	29 ± 6	28 ± 6	.76
Race, white	16 (70)	15 (71)	.89
Primipara	12 (52)	10 (48)	.76
Maternal education, y	15 ± 3	14 ± 3	.53
Public insurance	12 (52)	10 (48)	.76
Hemoglobin at admission, g/dL	11.7 ± 1.1	11.9 ± 1.1	.51
Lead level at admission, µg/dL	1.1 ± 0.4	1.0 ± 0.3	.38
Ferritin at admission, ng/mL	25.3 ± 26	18.8 ± 17	.34
Mode of delivery: vaginal	17 (74)	14 (67)	.60
Edinburgh Postnatal Depression Scale total score	3 ± 3	5 ± 5	.12
Parental Stress Index total score	51 ± 14	55 ± 16	.37
Infant			
Gestational age at birth, d, range	279.3 ± 8	277.8 ± 8	.54
Birth weight, g	3589 ± 521	3411 ± 430	.23
Male	12 (52)	12 (57)	.74
Cord-clamping time, s (includes UCM)	172 ± 188*	28 ± 76	.002
Cord-clamping time, s (without UCM) (n = 15, 20)	250 ± 190†	28.1 ± 78	<.001
RPBV, mL/kg	22.1 ± 8.5‡	27.2 ± 7.3	.05
Protocol violations	4 (17)	2 (10)	.45

*UCM*, umbilical cord milking.

Values are n (%) or mean ± SD.

* *P* < .01.

† *P* < .001.

‡ *P* < .05.

Table II shows no differences in hemoglobin, hematocrit, or other blood values at 4 months of age with analysis by intention to treat. However, infants who received DCC exhibited greater ferritin and log ferritin levels, and the absolute (relative) effect size was 31.1 (48%), 95% CI –59.7, –2.5. All ferritin levels were within normal range.^43^ Ferritin levels <40 occurred in 22% of the in the ICC group compared with 9% of the DCC group (*P* = .23). The mode of feeding was not different between groups and was not a significant predictor for ferritin. Thus, it was not included in a model for ferritin and VFm. None of the infants in either group received iron supplementation.

**Table II:** Clinical variables for infants with MRI (intention-to-treat)

Variables	DCC	ICC	*P* value
	(n = 23)	(n = 21)	
Neonatal			
Apgar scores, median (range)			
1 min	8 (3-9)	8 (2-9)	.77
5 min	9 (8-9)	9 (5-9)	.67
Cord hematocrit, %	43.7 ± 6	45.8 ± 5	.25
Cord ferritin, ng/dL	145 ± 92	141 ± 93	.89
BiliTool, high-risk zone (bilitool.org)	2 (9)	2 (10)	1.00
Peak total bilirubin, mg/dL	8.5 ± 4	9.1 ± 2	.56
Two-day hematocrit, %	57.6 ± 6*	53.1 ± 6	.01
Two-day hemoglobin, g/dL	19.1 ± 2	18.0 ± 2	.06
4-mo variables			
Hematocrit, %	34 ± 2.3	34 ± 2.4	.76
Hemoglobin, g/dL	11.7 ± 1.0	11.7 ± 0.7	.93
Ferritin, ng/dL	96.4 ± 58*	65.3 ± 32	.03
Log ferritin	4.4 ± 0.5*	4.1 ± 0.5	.03
Mean corpuscular volume, fL	81.4 ± 4.0	81.5 ± 3.7	.94
Transferrin, mg/dL	228 ± 31	239 ± 35	.28
Soluble transferrin receptor, mg/L	3.8 ± 0.9	3.8 ± 0.8	.93
C-reactive protein, mg/L	0.35 ± 0.4	1.0 ± 1.7	.08
Mullen Early Learning composite score	105.1 ± 8.7	103.5 ± 9.2	.55
Nonverbal composite score	120.5 ± 19.8	116.3 ± 21.0	.50
Verbal composite score	111.6 ± 21.5	109.2 ± 19.7	.70

Values are n (%), mean ± SD, or median (full range).

* *P* < .05.

There were no significant differences between groups on any of the other blood values examined (Table II). We found no significant differences in neurodevelopmental testing in the Mullen verbal and nonverbal developmental quotient composite scores or overall cognitive ability between the DCC and ICC groups (Table II). The values highlight that both groups fall within the normal range of Mullen scores and are within 1 SD of the standardized mean.

**Figure 2:**
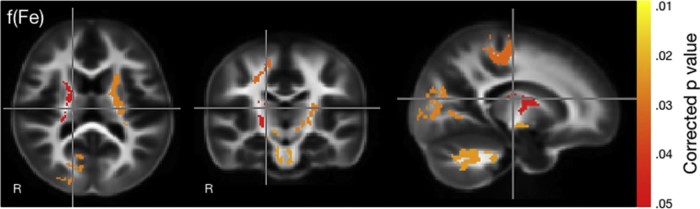
Correlation between myelin and ferritin at 4 months of age. Significance is indicated by the color scale on the *right* with *yellow* at *P* value of .01 and *red* indicating .05.

There were significant positive associations between VFm and 4-month blood ferritin levels (Figure 2). In particular, these associations were localized in regions of early developing white matter, including the right hemisphere cerebellar white matter, brain stem, parietal and occipital lobes, as well as the left and right anterior and posterior internal capsules. In all cases, greater levels of ferritin were associated with increased VFm. Controlling for sex did not affect the findings.

**Figure 3:**
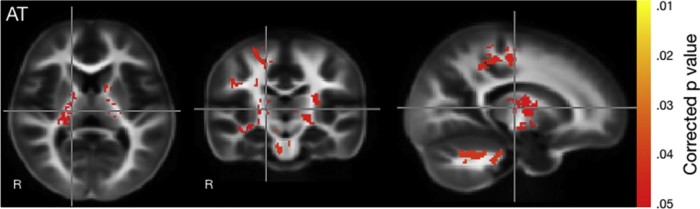
Group differences in myelin content between infants with DCC vs ICC by actual treatment. Significance is indicated by the color scale on the *right* with *yellow* at *P* value of .01 and *red* at a *P* value of .05. These colors represent areas in which myelin is greater in infants who had DCC compared with those who had ICC.

Dichotomous comparisons between infants in the DCC and ICC groups revealed infants exposed to DCC had significantly more myelin content in early myelinating areas than infants exposed to ICC. Analysis was completed using both intention to treat and actual treatment. Both analyses demonstrated significant differences, but actual treatment showed more robust differences in the various brain regions (Figure 3). Regions with increased myelin included the brain stem and cerebellum, left and right posterior arms of the internal capsule, and parietal lobe white matter. Controlling for sex did not yield any differences.

## Discussion

Infants who received a placental transfusion had greater ferritin levels at 4 months of age compared with those with ICC, as previously reported.^3^ In addition, these greater ferritin levels were associated with increased brain myelination at 4 months of age. Using mcDESPOT-derived VFm, a novel quantitative MRI measure of brain myelin content, we found that infants who received DCC had increased myelination at 4 months of age compared with those who received ICC. We observed significant VFm differences between infants receiving DCC and ICC, with infants receiving DCC having increased VFm in similar brain regions associated with the blood ferritin levels. Collectively, these results suggest a direct neurophysiological link between DCC and early myelin development, reinforcing and strengthening the literature that draws attention to the benefits of DCC in the newborn and supporting the previous finding that an endowment of iron-rich blood cells facilitated by placental transfusion is associated with increased iron storage and blood ferritin levels.^3^ This study extends the available evidence to show that increased ferritin levels are associated with greater brain myelin content at 4 months of age.

Beginning in the late second trimester and early third trimester, oligodendrocytes lay the groundwork for the lipid myelin bilayers that sheathe neuronal axons in a carefully orchestrated pattern that extends center-out and from posterior to anterior brain regions.^44^, ^45^ This process initiates within the brain stem and cerebellum, progresses to the cerebellum and internal capsules by the first postnatal month, and extends to parietal and occipital white matter between 4 and 6 months of age, before continuing its protracted developmental trajectory across the cortex.^31^, ^36^, ^46^ Over the first 2 postnatal years, myelination advances rapidly, with myelin present in nearly all brain areas by 9 months of age, and approximately 80% of adult levels reached by the end of year 2. An activity-driven process,^47^ the establishment and maintenance of the myelin sheath requires timed delivery of essential lipids and micronutrients, including iron.^23^, ^30^, ^48^ Significant associations between blood ferritin levels and VFm as well as VFm differences between infants with DCC and ICC were localized to these early developing brain regions, including the brain stem, cerebellar, parietal and occipital white matter, and the internal capsules. Our findings suggest that placental transfusion at birth may result in increased iron stores, represented by ferritin, and may help promote myelination in the first few months of life. This is particularly important, as myelinated axons facilitate rapid and efficient brain communication and messaging.^49^, ^50^ Future research examining whether these myelination differences between infants with DCC and ICC persist, become more extensive, or normalize over time will be important. Evaluation of the long-term consequences of DCC on infant brain development and other neurodevelopmental outcomes is planned. In this RCT, infants will return for MRI scans and neurodevelopmental testing at 12 and 24 months of age, providing the opportunity to continue to study such outcomes.

The early developing brain regions, ie, the internal capsules, differed between infants with DCC and ICC. These areas of the brain are essential to a wide variety of cognitive functions, including motor and sensory processing.^46^ Previous studies investigating neurobehavioral outcomes following DCC using neurodevelopmental testing only demonstrated improved scores in fine motor and the social domains in infants with DCC at 4 years of age, especially in boys,^51^ although no differences were seen at 4 and 12 months of age.^3^, ^52^ Our findings suggest that differences in myelin content may underlie neurodevelopmental differences between infants with DCC and ICC that appear later in childhood. The present study examined neurodevelopmental outcomes in infants at 4 months of age as this stage of infancy marks the onset of the most rapid period of myelin development.^46^ We observed no neurodevelopmental differences between the DCC and ICC groups at this early time. VFm differences between children with above-average and below-average cognitive ability do not present until early toddlerhood (1-2 years).^53^ Thus, neurodevelopmental gains resulting from DCC may not be observable until later in development. Assessment of the infants enrolled in our current RCT at 12 and 24 months of age will allow us to examine whether these differences manifest over time.

One potential mechanism underlying our findings of early myelination in infants with DCC and ICC may be related to iron. Iron is involved in myelinogenesis and is a necessary component for the maturation and function of the oligodendrocytes.^21^ Studies in animals have demonstrated that ID can lead to altered myelin lipid synthesis,^15^, ^54^ changes in myelin basic protein transcripts,^55^ and fundamental changes to the myelin-producing oligodendrocyte populations.^21^ ID can disrupt the trajectory of myelination growth and subsequently result in long-lasting myelin alterations.^13^ Our findings associating VFm and blood ferritin levels have not been reported previously. The increased iron stores afforded by increased red cell volume at birth facilitated by DCC appear to lead to increased infant myelination at 4 months of age. Myelin-producing oligodendrocytes, the predominant cell type containing iron, are composed of a mixture of ferritin subunits, which allows these cells to both store and use iron in the biosynthesis of cholesterol and lipids for myelin production.^21^ Thus, increased iron endowed through placental transfusion as measured by ferritin may enable oligodendrocytes to more rapidly accumulate iron and initiate and sustain myelination more quickly. However, this theory should be more specifically investigated with additional research in humans and animals. Nonetheless, our findings provide further evidence of an association between iron or ferritin and early brain myelination and may have important implications for clinical practice based on these underlying mechanisms.

This study used a 5-minute delay for DCC. When the study began in 2010, skin-to-skin care was adopted by the hospital as the standard of care for healthy infants born at term. We chose the 5-minute delay based on our pilot study,^56^ which showed that RPBV was significantly greater in infants placed skin-to-skin with ICC or a 2-minute delay compared with infants with a 5-minute delay or cord milking (×5). We wanted to obtain the maximum difference in placental transfusion between groups to optimize variances in the MRI results. One concern was that a delay of 5 minutes in this RCT resulted in a RPBV of 20 mL/kg, which was more residual blood than expected. It is also more than we found in our earlier pilot study, which yielded 11 mL/kg for infants born at term after 5 minutes. In addition, Yao reported 13.8 mL/kg of RPBV after a 3-minute delay with infants held below the level of the perineum, suggesting that placing the infant on the maternal abdomen slows the placental transfusion.^2^

Although we demonstrated greater ferritin levels at 4 months with DCC, the levels were lower than those in a study by Andersson et al, who reported a 3-minute delay but did not discuss placement of the infant.^3^ In a personal conversation, the lead author reported that the midwives held the infants below the level of the placenta for about 30 seconds as cord blood gases were obtained. Infants were then placed skin-to-skin. It is possible that the infants obtained more placental transfusion during those first 30 seconds.

Despite the findings of greater ferritin levels in the DCC group at 4 months, we found no differences in the hemoglobin and hematocrit levels. This finding is consistent with other studies in infants born at term,^3^, ^57^ suggesting that hemoglobin and hematocrit levels do not adequately represent the infant's body iron stores. Yet, ferritin levels are not assessed routinely at 4 months. Thus, most pediatric providers rely on the hemoglobin and hematocrit to reflect iron status.^58^

Although the current study suggests DCC results in better VFm outcomes in infants at 4 months of age, and mcDESPOT has shown qualitative agreement with myelin histology,^59^, ^60^ future studies are needed to quantitatively validate mcDESPOT measures. Nonetheless, the extant literature using mcDESPOT^31^, ^32^, ^33^, ^37^, ^38^ provides confidence that mcDESPOT-derived VFm measurements are sensitive to myelin content.

Placental transfusion via DCC facilitates a transfer of residual iron-rich placental blood and increases iron stores without adverse effects. Our findings show that infants who received a placental transfusion have increased myelin content at 4 months of age compared with infants who received ICC, adding to a growing number of studies that describe the benefits of DCC. Moreover, given that DCC is a feasible, low-tech, no-cost approach, it has the potential to have widespread impact on early life development. Future studies examining the long-term effects of DCC on child development would be important, but the ethical concerns regarding comparisons to ICC are to be considered.

## Appendix

### Supplementary Materials

#### Methods: Blood Samples

The complete blood count samples were collected in a 0.5-mL EDTA tube (BD Microtainer, Franklin Lakes, New Jersey) and then analyzed using an automated hematology analyzer (Sysmex XN 3000; Sysmex America Inc, Lincolnshire, Illinois). The iron indices and C-reactive protein were collected in a 0.5-mL serum separator tube (BD Microtainer) and analyzed with a clinical chemistry analyzer (Architect ci4100, Abbott Laboratories, Abbott Park, Illinois). All samples, except ferritin, were processed at Women & Infants Hospital. Ferritin was processed at the Mayo Medical Laboratories (Rochester, New York) using an immunoassay system (Beckman Coulter Unicel DXL 800; Beckman Coulter Inc, Brea, California).

#### MRI Data Acquisition and Processing

Parents were contacted to schedule the 4-month MRI. Children were brought to the MRI center either at nap or bedtime. Special sleep rooms were provided for parents to get the infant to sleep. When sleeping, the infant was placed securely on an MRI-compatible cart and transported to the MRI scanner. Parents (after appropriate screening) were invited to stay with the infant during the MRI. The MRI scan lasted approximately 30-45 minutes once the infant was asleep. If an infant was unable to fall asleep or to return to sleep after waking, the MRI examination was rescheduled. Within 1 week after a successful MRI scan, children were scheduled for developmental testing.

Measures of brain myelin content were acquired with the mcDESPOT MRI technique. mcDESPOT is a multicomponent relaxometry technique that decomposes the measured MRI signal into contributions from myelin and nonmyelin water-based on the unique relaxation properties of each of these water pools.^1^, ^2^, ^3^, ^4^ Unlike traditional multicomponent relaxometry techniques,^1^, ^2^ mcDESPOT uses rapid and time-efficient gradient echo sequences, acquired over a range of flip angles, to quantify the relaxation characteristics of multicompartment water pools.^5^, ^6^ Specifically, the mcDESPOT protocol included 8 T_1_-weighted spoiled gradient-recalled echo (SPGR) and 16 T_1_/T_2_-weighted balanced steady-state free precession (bSSFP) images acquired over multiple flip angles.^5^, ^6^ Two inversion-prepared (IR)-SPGR images additionally were acquired for correction of radio-frequency (B_1_) inhomogeneities, and bSSFP images were acquired with 2 phase cycling patterns (φ = 180° and 0°) for correction of main magnetic field (B0) inhomogeneities.^7^ Choice of scan acquisition parameters for the mcDESPOT protocol have been optimized according to the relaxation characteristics at various stages of infancy and early childhood.^8^ Specific acquisition parameters of the SPGR, bSSFP, and IR-SPGR scans used in the current study are as follows:

*SPGR:* repetition time (TR) = 12 milliseconds; echo time (TE) = 5.8 milliseconds; flip angles (α) = [2, 3, 4, 5, 7, 9, 11, 14] degrees; receiver bandwidth  = 350 Hz/voxel; and 6/8 partial k-space in the phase and slice-encode directions.

*bSSFP:* TR = 10 milliseconds; TE = 5 milliseconds; α = [9, 14, 20, 27, 34, 41, 56, 70]; bandwidth = 350 Hz/voxel; 6/8 partial k-space in the phase and slice–encode directions.

*IR-SPGR:* TR = 12 milliseconds; TE = 5.8 milliseconds; inversion times  = [600, 950] milliseconds; α = 5 degrees; 6/8 partial k-space in the phase-encode directions. Half the resolution in the slice direction.

All data were acquired from each participating 4-month-old infant on a Siemens Tim Trio 3 Tesla scanner (Siemens) with a 12-channel head radiofrequency array. To help the children sleep during the scan, acoustic noise levels were minimized by reducing imaging gradient slew rates and peak values. Additional passive sound attenuation was achieved with a sound-insulating bore liner (Ultra Barrier HD Composite; American Micro Industries, Chambersburg, Pennsylvania) and MiniMuff ear pads. Electrodynamic and sound-attenuating headphones (MR Confon GmbH, Magdeburg, Germany) also were used and provided constant white noise throughout the duration of the scan.^9^

Following successful acquisition, image data were inspected visually for motion-related image artifacts (eg, edge blurring and ghosting). Each participant's SPGR, bSSFP, and IR-SPGR images were then linearly coregistered to account for subtle head movement^10^ and nonbrain (ie, skull) signal was removed.^11^ SPGR and IR-SPGR images were used to estimate the flip angle correction map.^12^ VFm values were calculated at each image voxel by fitting the SPGR and bSSFP data to a multicomponent relaxometry model of 3 microstructural water compartments: intra/extra-axonal water, myelin-associated water, and nonexchanging free water.^6^

For correlation analysis and group comparisons, individual VFm maps were nonlinearly aligned to a common study template^8^ using a fully 3-dimensional image registration approach.^13^ Before statistical analyses, aligned VFm data were smoothed with a modest 4-mm full-width-at-half-maximum 3D Gaussian kernel to account for residual registration inaccuracies.^14^
